# Turkish version of the ‘food and nutrition literacy questionnaire for Chinese school-age children’ for school-age adolescents: a validity and reliability study

**DOI:** 10.1186/s12889-023-16732-2

**Published:** 2023-09-16

**Authors:** Özge Mengi Çelik, Merve Seyda Karacil Ermumcu, Caner Ozyildirim

**Affiliations:** 1 Department of Nutrition and Dietetics, Gulhane Faculty of Health Sciences, University of Health Sciences, Ankara, Türkiye; 2 Department of Nutrition and Dietetics, Faculty of Health Sciences, Akdeniz University, Dumlupinar Boulevard International Relations Office Rectorate 6th Floor 07058 Campus, Antalya, Türkiye

**Keywords:** Nutrition literacy, Food literacy, Children, Nutrition, Diet

## Abstract

**Background:**

In this study, it was aimed to examine the psychometric characteristics of the scale named ‘Food and Nutrition Literacy Questionnaire for Chinese School-age Children (FNLQ-SC)’ in Turkish school age adolescents.

**Methods:**

The research was carried out with 341 school-age adolescents aged 10 to 17 years. The Cronbach’s α coefficient was used to evaluate internal consistency reliability and the test–retest method was applied. The construct validity was assessed by exploratory factor analysis (EFA) and confirmatory factor analysis (CFA), and the content validity was assessed by the Pearson correlation coefficient.

**Results:**

EFA indicated that the Turkish version of FNLQ-SC had three factorial structures that accounted for 42.0% of the total variance. The overall Turkish version of FNLQ-SC questionnaire had acceptable internal consistency (Cronbach’s α = 0.679). The dimensional structure obtained in the EFA was controlled by CFA and the three-factor model showed acceptable goodness-of-fit indices (χ2/df = 1.924, RMSEA = 0.052, CFI = 0.864, GFI = 0.949). The Pearson correlation coefficients between each dimension and the overall questionnaire ranged from 0.300 to 0.842. Multiple linear regression analysis indicated that age, gender, grade of class, being an only child and discussing nutrition information with families had an effect on food and nutrition literacy (R^2^ = 0.312; *p* < 0.001).

**Conclusion:**

The Turkish version of FNLQ-SC has good reliability and construct validity to assess the food and nutrition literacy of Turkish school age adolescents.

## Background

Food literacy and nutrition literacy are important factors that support healthy eating habits. Nutritional literacy is defined as the degree to which individuals have the capacity to acquire, process and understand nutritional knowledge and skills, and the ability to make appropriate decisions about nutrition. In other words, it is the degree to which individuals read and understand nutritional information in order to make appropriate nutritional decisions and gain nutritional skills. Today, a global change is taking place in the food systems. Access to unhealthy foods that are highly processed, low in nutrients and high in energy density is easier than accessing nutritious and healthy foods, which negatively affects diet quality. In this process, the concept of food literacy has gained an important place. Food literacy is expressed as the ability of individuals to make choices in the changing food environment to maintain their diet quality and healthy nutrition. Having a high level of food and nutritional literacy provides the necessary skills and abilities to make the right decision in the current food environment [1–5].

Food and nutritional literacy ensures that healthy eating practices are supported and sustained. Starting from the pre-school period, supporting food and nutrition literacy in individuals is very important in terms of healthy food choices and nutritional habits. Nutritional habits, starting from the pre-school period and continuing in the adolescence period, are important in terms of non-communicable chronic diseases. Unhealthy diets and insufficient physical activity in adolescence increase the risk of non-communicable diseases by causing excessive weight gain and obesity. This period is a critical period for both childhood and adulthood, and is the best time to develop positive health behaviors that can be sustained throughout life. During this period, energy and nutrient requirements increase. Inadequate intake of macro and micronutrients, unhealthy food choices and dietary habits adversely affect the health, nutritional status and physical and mental development of adolescents [6–9]. In addition, healthy eating behaviors and a high level of food and nutrition literacy are closely related to academic success in school-age adolescents [10]. In Turkiye, thinness, obesity, vitamin deficiencies, dental caries and anemia are among the most common problems associated with malnutrition [11]. Just at this stage, the importance of food and nutritional literacy in adolescents comes into play. Low food and nutrition literacy hinders the provision of dietary diversity and nutrient adequacy [12].

The acquisition of nutritional literacy skills should start at the earliest possible period in order to create a health culture in the society and to ensure the permanence of healthy behaviours. In addition to nutritional knowledge, skills, knowledge and capacity related to nutrition are also important. There is a need for valid and reliable scales to be used in practices aimed at determining food and nutrition literacy in the whole society and especially in risk groups. Although research in this field is increasing, there is no recognised method that can be used to measure food and nutrition literacy in Turkish children and adolescents. In this study, it was aimed to examine the physiometric characteristics of the scale named ‘Food and Nutrition Literacy Questionnaire for Chinese School-age Children’ in Turkish school age adolescents. We think that the validity and reliability of this scale in Turkish is important in the following ways: 1) This scale takes different dimensions into consideration when measuring nutrition literacy (knowledge and understanding of food and nutrition, access to and planning for food, selecting food, preparing food and eating). 2) This scale provide targets for further nutrition education and intervention 3)This scale focuses not only on the ability to access and understand nutritional information but also on the ability to judge and apply nutritional information, and the ability to communicate and act on that information. We thought that this study will fill the gap in the literature and lead to studies on food and nutrition literacy in adolescents.

## Methods

### Participants

In this methodological study, the cross-cultural adaptation of the scale was performed by following the guidelines provided by Beaton et al. [13]. Afterwards, the psychometric properties of the food and nutrition literacy scale were examined. For scale adaptations, it is recommended to choose a sample of at least 5–10 in the number of scale items [14]. Given the number of items on the scale and for taking good results, the researchers planned to include at least 250 adolescents. This study was conducted with 341 school-age adolescents aged 10–17 years between June and November 2022.

### Ethical considerations

Before starting the study, ethics approval with the decision number 347 dated 11.05.2022 was obtained from the Clinical Research Ethics Committee of Akdeniz University Faculty of Medicine. For cultural adaptation, written permission was taken from the responsible author of the scale. Parental written consent was obtained on behalf of all participants below the age of 16. Adolescents were informed regarding the study’s purpose and their written and verbal consent were obtained before the participation. The adolescents who participated in the research were asked to fill out the informed volunteer consent form, which explained the aim of the study. Adolescents were informed that they could leave the study whenever they wished in line with stipulations of Declaration of Helsinki Ethical principles.

### Data collection tools

Data were collected with a questionnaire form and Food and Nutrition Literacy Questionnaire for Chinese School-age Children (FNLQ-SC). The demographic characteristics (age, gender, educational status of students and parents, number of children in the family, economic situation), anthropometric measurements and food consumption of the students were questioned. Dietary quality of adolescents was calculated using food consumption.

### Anthropometric measurements

Body weight was taken with a calibrated scale and height was measured with the stadiometer in accordance with their techniques [15, 16]. The body mass index (BMI) value was calculated by dividing the body weight by the square of the height and evaluated according to World Health Organization (WHO) growth standards [17]. BMI for age Z scores were computed using the WHO AnthroPlus software (version 1.0.4). Waist circumference was taken with a non-stretchable tape in accordance with the measurement technique [15].

### Food consumption and diet quality

The adolescents’ 24-h dietary recalls were taken using the Food and Nutrition Photo Catalog [18]*.* Dietary data were evaluated using the Nutrition Information System (BeBiS) which is a food analysis software program. The quality of dietary intake was assessed using the Healthy Eating Index (HEI)-2015. Index contains 13 dietary components. Nine adequacy components (those recommended for inclusion in a healthy diet) contain whole grains, dairy, total fruits, whole fruits, total protein foods, total vegetables, greens and beans, seafood and plant proteins, and fatty acids. Four moderation components (those that should be consumed sparingly) contain added sugars, refined grains, saturated fats and sodium. The HEI total score is the sum of the scores obtained from all the components. A total score of ≤ 50 was described as “poor diet quality”, scores of 51–80 considered “needs improvement” and scores > 80 indicated ‘good diet quality’ [19].

### Food and Nutrition Literacy Questionnaire for Chinese School-age Children (FNLQ-SC)

The scale was developed by Liu et al. [20] in 2021 to evaluate the level of food and nutrition literacy of school-age children. The scale consisted of 50 items (questions), 19 core components and 5 dimensions, these dimensions were knowledge and understanding of food and nutrition, access to and planning for food, selecting food, preparing food and eating. The dimensions of knowledge and understanding, access to and planning for food, selecting food, preparing food and eating included 15, 5, 5, 10, 15 questions respectively The questions included 5-point Likert-type questions (‘I am concerned about nutrition and health information: never, seldom, sometimes, usually, always’), choice questions (‘Which of the following snacks is healthier?’), and fill-in-the-blank questions (‘Fill in your height and weight.’). Each question was scored 2 points and maximum score of scale was 100. The Cronbach's α coefficient for overall questionnaire was 0.698 and for the five dimensions (knowledge and understanding, access to and planning for food, selecting food, preparing food, eating), were 0.452, 0.300, 0.244, 0.148, and 0.436, respectively.

## Data analysis

While IBM AMOS version 26.0 was used for confirmatory factor analyses, IBM SPSS Statistics for Windows version 26.0 was used for the remaining statistical analyses. A p-value of < 0.05 was considered statistically significant. The reliability and validity were analyzed on the basis of components, not the questions, because some questions assessed more than one component. The relationships between the variables are given with the Pearson correlation coefficient. Chi-square analysis was used to compare qualitative data and detect differences between groups. Mean differences between groups were assessed by independent t-test. Regression analysis was performed for prediction of food and nutrition literacy. One-way ANOVA test was used to evaluate whether there was a statistically significant difference between the means of independent groups.

### Construct validity

The internal construct validity of the Food and Nutrition Literacy Questionnaire for Turkish School-age Children (FNLQ-TSC) was assessed by exploratory factor analysis (EFA) and confirmatory factor analysis (CFA). The sufficiency of the sample size and suitability of the data for the factor analysis were examined by Kaiser Meyer Olkin (KMO) Test and Bartlett’s test, respectively. The factors were retained based on eigenvalues of more than one. Items with a factor loading of 0.40 and above were selected for the relevant factor. If the item was 0.40 and above in two or more factors, attention was paid to keep the difference between the two loads 0.1. Otherwise, that item was eliminated because it was cross-loading [21]. To evaluate the factors’ goodness of fit, the ratio of the Chi-square test of model fit to the degrees of freedom (χ2/df) [values of five or less], the Comparative Fit Index (CFI: > 0.90 acceptable and > 0.95 excellent), and the Root Mean Square Error of Approximation (RMSEA: < 0.08 acceptable and < 0.05 excellent) were used [22]. External construct validity of the Food and Nutrition Literacy Questionnaire for Turkish School-age Children (FNLQ-TSC) was assessed by hypothesis testing (convergent validity) [23]. For the process of convergent validity, it was used the Spearman’s correlation coefficients (rho) for expected associations of the FNLQ-TSC with HEI-2015 total score.

### Discriminant validity

Participants were divided into three groups using the HEI-2015 total score to determine whether the scale discriminated [24].

### Reliability

To determine the reliability of the FNLQ-TSC, internal consistency was evaluated by the calculation of Cronbach’s α coefficient [25]. The Cronbach’s α value of ≥ 0.70 is considered acceptable, 0.80 good, and < 0.60 poor or unacceptable [26]. Also, the scale was repeated in 30 adolescents after an interval of 4 weeks for test–retest reliability.

## Procedure

### The translation process

The authors obtained permission via e-mail from the responsible author of the scale to evaluate the Turkish psychometric properties of the scale named ‘Food and Nutrition Literacy Questionnaire for Chinese School-age Children’. The cross-cultural adaptation of the scale was performed by following the guidelines provided by Beaton et al.[13]. The translation of the scale into Turkish was completed in six steps. These steps were translation, synthesis, back-translation, expert committee review, pretesting and final version. In first step, five health professionals who were familiar with the terminology of the translated scale and had experience in interviewing/data collection took part in the translation process of the scale. Five experts translated the scale from English to Turkish. In the second step, after the translation was finished, the researchers evaluated the semantic, idiomatic, conceptual, linguistic, and contextual differences by comparing different translations to create a single Turkish form. In next step, health professionals performed the back translation of the scale from Turkish into English. In step four, an expert committee developed the prefinal Turkish version of the scale. Then, the scale was tested on 20 adolescents. After the pretesting, it was determined that all participants rated all items in the scale as ‘clearly understandable’, which indicated that the scale was appropriate for this population. The final version of the scale was approved in the last step.

### Specialist opinions and content validity

It is recommended to get opinions from at least 3–20 experts for content validity [14]. In this study, opinions were obtained from 10 academicians who are experts in food and nutrition literacy for content validity, scale-level content validity index (S-CVI) and item–content validity index (I-CVI) were calculated using Polit and Back's content validity index [27]. Experts were asked to rate the translated scale in Turkish and the original version between 1–4 (1 = very little change required, 2 = little change required, 3 = appropriate and 4 = very convenient) to evaluate the suitability of the items of the scale [14]. S-CVI and I-CVI were calculated separately for each item of the scale. Items with 1 and 2 points on the scale items were changed according to the experts’ recommendation [14]. I-CVI ranged from 0.92 to 0.99, and S-CVI was 0.96, which was coherent.

### Preliminary test

The World Health Organization recommends that back-translation should be done after taking expert opinion [28]. In this study, back translation was carried out by two translators after receiving expert opinions. The Turkish form of the scale was translated into English by two linguists who knew Turkish and English well [29]. After back translation, the scale was compared with its original version, necessary adjustments were made, and the scale was made ready for preliminary test. It was suggested that the scale be applied to 20 to 30 participants who had similarities with the sample population but were not included in the study sample [30]. In this context, the scale was applied to 30 school-age adolescents, and this data were not included in the current study. As a result of the pilot application, the comprehensibility of each item was evaluated. There was no negative feedback from adolescents. Reliability and validity analyses of the scale were performed.

## Results

### Participant characteristics

A total of 341 participants (195 female, 146 male) with a mean age of 13.6 ± 2.5 years were enrolled in the study. 59.8% of the students were studying in the 4th to 8th grades, 40.2% of them were in the 9th to 12th grades. Almost all of the students (94.7%) were living at home. 10.3% of the students were only child. The income of most families (49.9%) was equal to their expenses. Most of the students' mothers (41.3%) had a low educational level, and most of the fathers of the students (36.4%) had a high education level. 18.2% of the students stated that they received education about nutrition at school. In addition, the majority of the students (51.9%) stated that they discussed the information about nutrition with their families. 60.4% of the students had normal body weight (Table 1).

**Table 1:** Demographic characteristics of participants

**Variables**	
**Gender**	
Male	146 (42.8%)
Female	195 (57.2%)
** Age (years)**	13.6 ± 2.5
**Grade of the class**	
4–8	204 (59.8%)
9–12	137 (40.2%)
**The place of residence**	
at home	323 (94.7%)
in the dormitory	18 (5.3%)
**Only child**	
Yes	35 (10.3%)
No	306 (89.7%)
**Income status of the family**	
Income more than expenses Income equal to expenses	91 (26.7%) 170 (49.9%)
Income less than expenses	80 (23.5%)
**Mother's educational level**	
Low education	141 (41.3%)
Medium education	109 (32.0%)
High education	91 (26.7%)
**Father's educational level**	
Low education	104 (30.5%)
Medium education	113 (33.1%)
High education	124 (36.4%)
**School nutrition education**	
Yes	62 (18.2%)
No	279 (81.8%)
**Discussion nutrition information with families**	
Yes	172 (51.9%)
No	164 (48.1%)
**BMI (kg/m**^**2**^**)**	20.1 ± 3.5
**BAZ classification**	
Thinness (< -1 SD)	50 (14.7%)
Normal (≥ -1 + 1 SD)	206 (60.4%)
Overweight (> 1 SD)	58 (17.0%)
Obesity (> 2 SD)	27 (7.9%)
**Waist circumference (cm)**	69.1 ± 13.0

*n* = 341, Low education < High school graduate, Medium education = High school graduate, High education ≥ University graduate, *BMI* Body mass index, *BAZ* BMI for age Z score

### Construct validity

**Fig. 1:**
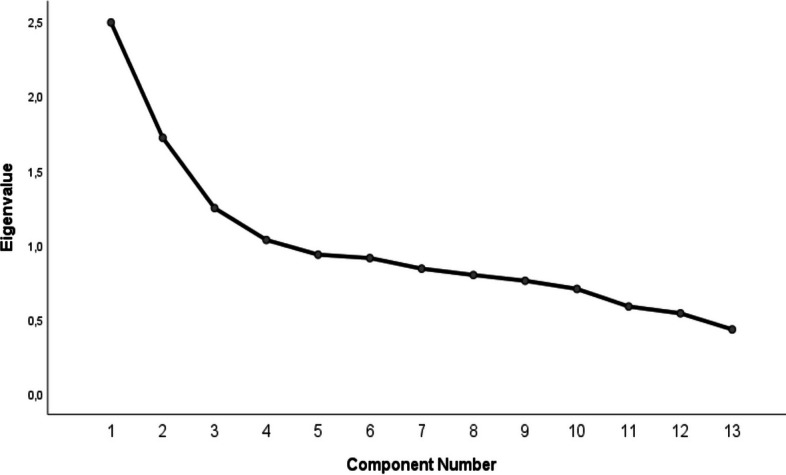
Scree plot of the exploratory factorial analysis of the FNLQ-TSC

EFA was perfomed to determine the structure of the FNLQ-TSC. The KMO and Bartlett’s sphericity test results revealed that the sample size was sufficient (KMO = 0.715), and the items were appropriate (Bartlett’s test of sphericity: χ2 = 794.416, *p* < 0.001) for the factor analysis. EFA indicated that the FNLQ-TSC had three factorial structures that accounted for 33.14% of the total variance. However, when the factor loading values of the items were examined, it was determined that six components (component 2, 7, 9, 10, 13, and 16) loaded on more than one factor and the difference between these factor loading values was less than 0.10 [31]. Therefore, these components were excluded from the scale by considering them as overlapping components, and the EFA analysis was re-run. In a similar way, the sample size was sufficient (KMO = 0.678), and the items were suitable (Bartlett's test of sphericity: χ2 = 490.870, *p* < 0.001) for the factor analysis. EFA indicated that the FNLQ-TSC had three factorial structures (Fig. 1) that accounted for 42.0% of the total variance. The first factor consisted of 6 components (components 3, 4, 11, 14, 14, 17, and 18), which discerned knowledge of food and nutrition and accounted for 19.2% of the common variance. While the second factor (13.2% of the common variance) consisted of 3 components (components 5, 6, and 12) which discerned understanding and planning for food, the third factor (9.6% of the common variance) consisted of 4 components (components1, 8, 15, and 19) which discerned food selection and awareness (Table 2).

**Table 2:** Exploratory Factor Analysis by principal components of the FNLQ-TSC

	**EFA factor loading**		
**Component**	**Factor 1**	**Factor 2**	**Factor 3**
1			0.639
3	0.449		
4	0.723		
5		0.538	
6		0.837	
8			0.460
11	0.581		
12		0.712	
14	0.504		
15			0.731
17	0.611		
18	0.449		
19			0.627
Eigenvalues	2.494	1.719	1.248

**Fig. 2:**
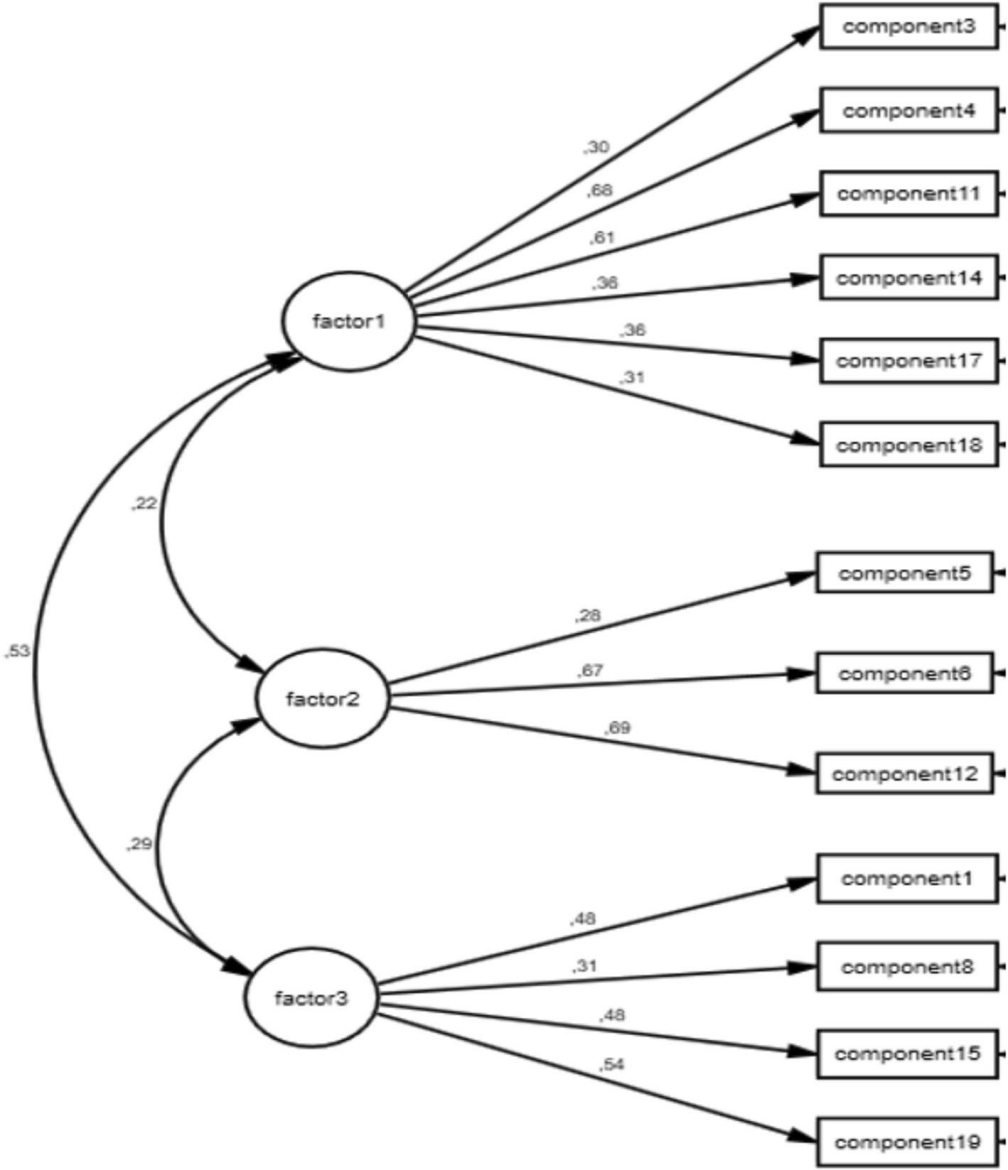
Three -factor model of the FNLQ-TSC

The dimensional structure of the FNLQ-TSC obtained in the EFA was controlled by CFA. The three-factor model (Fig. 2) showed acceptable goodness-of-fit indices. (χ2/df = 1.924, RMSEA = 0.052, CFI = 0.864, GFI = 0.949).

### Discriminant validity

The discriminant validity of the FNLQ-TSC is shown in Table 3. A statistically significant difference was not found between the FNLQ-TSC total scores of individuals according to diet quality (*p* > 0.05). Therefore, the scale was not able to discriminate levels of diet quality.

**Table 3:** Evaluation of FNLQ-TSC scores of individuals according to diet quality

Diet quality	FNLQ-TSC total score	*p*
Poor diet	42.2 ± 7.15	0.687
Needs improvement	43.2 ± 7.52	
Good diet	40.8 ± 13.78	

### Hypothesis testing- convergent validity

There was a moderate positive and statistically significant relationship between the FNLQ-TSC total score and HEI-2015 total score (*r* = 0.518, *p* < 0.001) (Table 4). A positive, statistically significant and strong correlation was observed between the test–retest total scores of the FNLQ-TSC (*r* = 0.766, *p* < 0.001) (Table 4).

**Table 4:** Correlation between total score of the FNLQ-TSC and HEI-2015

	**r**	***p***
FNLQ-TSC and HEI-2015	0.518	< 0.001*
FNLQ-TSC and FNLQ-TSC a month later	0.766	< 0.001*

Pearson correlation

*p< 0.05

### Content validity

The Pearson correlation coefficients between different dimensions ranged from 0.075 ~ 0.244. The correlation coefficients between each dimension and the overall questionnaire ranged from 0.300 to 0.842, especially the coefficients of factor-1 and factor-3, were more than 0.6, which showed a strong correlation with the overall questionnaire (Table 5).

**Table 5:** Pearson correlation coefficient among dimensions of FNLQ-TSC

Dimensions	Factor 1	Factor 2	Factor 3	FNLQ-TSC total score
Factor 1	-	*r* = 0.075 *p* = 0.170	*r* = 0.244 *p* < 0.001*	r = 0.842 *p* < 0.001*
Factor 2	*r* = 0.075 *p* = 0.170	-	*r* = 0.105 *p* = 0.052	*r* = 0.300 *p* < 0.001*
Factor 3	*r *= 0.244 *p* < 0.001*	*r* = 0.105 *p* = 0.052	-	*r* = 0.694 *p* < 0.001*

*p< 0.05

### Reliability

Table 6 displays mean FNLQ-TSC component scores, component-total correlation, and Cronbach’s a finding if an item is removed. The Cronbach’s α values for internal consistency were 0.679, 0.581, 0.551, and 0.350 for the FNLQ-TSC total score, factor-1, factor-2, and factor-3, respectively. Based on these values, the overall FNLQ-TSC questionnaire had acceptable internal consistency, however, the Cronbach’s α coefficient of dimensions was low, ranging from 0.350 to 0.581.

**Table 6:** Mean scores corrected component-total correlations and Cronbach’s a if item deleted results for the FNLQ-TSC

Component	Mean ± SD	Corrected component-total correlation	Cronbach’s a if component deleted
1	0.53 ± 0.53	0.316	0.562
3	1.06 ± 1.00	0.216	0.563
4	4.68 ± 2.24	0.418	0.505
5	1.24 ± 0.63	0.134	0.590
6	0.61 ± 0.68	0.176	0.571
8	1.49 ± 0.62	0.234	0.566
11	4.64 ± 1.80	0.430	0.505
12	0.98 ± 0.65	0.246	0.565
14	1.13 ± 0.99	0.238	0.560
15	14.93 ± 2.84	0.218	0.605
17	1.34 ± 0.94	0.245	0.560
18	6.02 ± 1.58	0.239	0.558
19	3.97 ± 1.08	0.358	0.540

### Assessing food and nutrition literacy and its related factors in school-age adolescents

There were higher food and nutrition literacy among school-age adolescents who were female, had a higher grade, stayed at home, were not an only child, had a high father's education level, discussed nutrition information with their family, and were normal weight (Table 7).

**Table 7:** Evaluation of food and nutritional literacy according to some variables in school-age adolescents

Variables	Total	Factor 1	Factor 2	Factor 3
Total	42.6 ± 7.29	18.9 ± 5.1	2.8 ± 1.4	20.9 ± 3.7
**Gender**				
Male	40.9 ± 7.1^a^	18.0 ± 5.2^a^	2.2 ± 1.3^a^	20.6 ± 3.6
Female	43.9 ± 7.1^b^	19.5 ± 5.0^b^	3.2 ± 1.3^b^	21.1 ± 3.7
**Grade of the class**				
4–8	41.0 ± 7.5^a^	17.5 ± 5.4^a^	2.5 ± 1.3^a^	21.0 ± 3.7
9–12	44.9 ± 6.2^b^	20.9 ± 3.8^b^	3.2 ± 1.4^b^	20.7 ± 3.6
**The place of residence**				
at home	42.8 ± 7.2^a^	19.1 ± 4.9^a^	2.8 ± 1.4	20.9 ± 3.7
in the dormitory	38.8 ± 7.3^b^	14.7 ± 7.4^b^	3.1 ± 1.5	20.9 ± 3.7
**Only child**				
Yes	39.0 ± 8.4^a^	15.7 ± 6.2^a^	2.5 ± 1.2	20.7 ± 4.1
No	43.0 ± 7.1^b^	19.2 ± 4.8^b^	2.8 ± 1.4	20.9 ± 3.6
**Income status of the family**				
Income more than expenses	44.3 ± 5.7	20.2 ± 3.5^a^	2.8 ± 1.5	21.3 ± 3.6
Income equal to expenses	42.9 ± 7.3	19.0 ± 5.2	2.8 ± 1.3	21.1 ± 3.7
Income less than expenses	40.1 ± 8.0	17.1 ± 5.9^b^	2.9 ± 1.3	20.1 ± 3.5
**Mother's educational level**				
Low education	41.0 ± 7.5	17.6 ± 5.8	2.9 ± 1.5	20.5 ± 3.5
Medium education	42.5 ± 7.0	18.9 ± 4.5	2.7 ± 1.3	20.8 ± 3.7
High education	45.2 ± 6.6	20.7 ± 4.1	2.8 ± 1.4	21.6 ± 3.7
**Father's educational level**				
Low education	40.5 ± 7.5^a^	17.0 ± 6.1	3.0 ± 1.4	20.4 ± 3.3
Medium education	42.0 ± 6.9^b^	19.0 ± 4.5	2.7 ± 1.4	20.3 ± 3.6
High education	44.9 ± 6.8^c^	20.2 ± 4.2	2.8 ± 1.4	21.8 ± 3.7
**School nutrition education**				
Yes	43.3 ± 8.1	19.2 ± 5.5	2.6 ± 1.5	21.5 ± 3.5
No	42.5 ± 7.1	18.8 ± 5.1	2.8 ± 1.4	20.7 ± 3.6
**Discussion nutrition information with families**				
Yes	44.8 ± 6.6^a^	20.4 ± 4.1^a^	2.8 ± 1.3	21.6 ± 3.7^a^
No	40.2 ± 7.2^b^	17.2 ± 5.6^b^	2.8 ± 1.5	20.2 ± 3.5^b^
**BAZ classification**				
Thinness (< -1 SD)	40.6 ± 6.3	17.0 ± 4.9	2.8 ± 1.2	20.8 ± 3.2
Normal (≥ -1 + 1 SD)	44.5 ± 6.8^a^	20.2 ± 4.6^a^	2.9 ± 1.4	21.4 ± 3.5
Overweight (> 1 SD)	40.0 ± 6.9	17.1 ± 5.3	2.7 ± 1.4	20.1 ± 3.6
Obesity (> 2 SD)	37.7 ± 8.2^b^	15.9 ± 5.5^b^	2.6 ± 1.4	19.1 ± 4.3

a, b, c indicate significant differences among groups (*p* < 0.05)

When the factors that could affect the total score of the FNLQ-TSC were evaluated with linear regression analysis, the model was deemed important (R^2^ = 0.312; *p* < 0.001). It was determined that age, gender, grade of class, being an only child and discussing nutrition information with families had an effect on food and nutrition literacy (*p* < 0.05) (Table 8).

**Table 8:** Multiple linear regression analysis of food and nutrition literacy-related factors among school-age adolescents

	**FNLQ-TSC total score**		
**Model**	***Beta***	**t**	***p***
Age (years)	0.191	2.008	0.045*
Gender	-0.133	-2.849	0.005*
Grade of the class	0.472	4.970	< 0.001*
The place of residence	-0.002	-0.042	0.967
Only child	-0.115	-2.459	0.014*
Income status of the family	0.058	1.184	0.237
Mother's educational level	0.089	1.570	0.117
Father's educational level	0.104	1.770	0.078
School nutrition education	0.068	1.466	0.144
Discussion nutrition information with families	0.228	4.820	< 0.001*
		**R**^**2**^ = 0.312; p < 0.001*	

*Variable values* Gender (Male = 1, Female = 0), Place of residence (at home = 0, in the dormitory = 1), Only child (Yes = 1, No = 0), Income status of the fasmily (Income less than expenses = 1, Income equal to expenses = 2, Income more than expenses = 3), Education level of fathers and mothers (Low education = 1, Medium education = 2, High education = 3), School nutrition education (Yes = 1, No = 0), Discussion nutrition information with families (Yes = 1, No = 0)

*p < 0.05

## Discussion

Food and nutrition literacy defined as a collection of interrelated knowledge, skills and behaviors required to plan, manage, select, prepare and eat foods to meet requirements and determine food intake [20]. Today, healthy dietary behavior describes by nutrition literacy or food literacy. Some researchers have focused on the ability to use food labels effectively [32, 33]. Building these skills and knowledge at a young age is important for skill retention, confidence in food practices and supporting lifelong healthy eating habits [2]. Measuring food and nutrition literacy is a new topic. Existing food and nutrition literacy tools tend to emphasise literacy, numeracy skills and nutrition knowledge, especially in adults. There are limited tools to identify food and nutrition literacy for children and adolescents [2]. Carrol et al. [2] identified tools to assess food and nutrition literacy in children and adolescents and evaluated their psychometric properties in a systematic review. Twelve instruments were included in the study, 6 of which had subscales with either poor or questionable internal consistency scores. Therefore, this suggests that further adaptations may be needed to improve consistency among these instruments [2]. Though research in this area is growing, progression is limited by the lack of an accepted method to measure food and nutrition literacy especially in adolescents. This study was conducted to establish the validity and reliability of the Turkish version of FNLQ-SC which was originally developed by Liu [20]. In Turkiye, there is no scale used to evaluate food and nutrition literacy in school-age adolescents. It is thought that this study will fill the gap in the literature and lead to studies on food and nutrition literacy in adolescents.

There are different tools such as Child Food Insecurity Experiences Scale [34], Food and Nutrition Literacy Questionnaire for Chinese School-age Children [20], Food and Nutrition Literacy Assessment Tool [35], Food Literacy Instrument [36] that assess food insecurity and nutrition literacy in children. The level of food and nutrition literacy is one of the ways to understand the reasons for the nutrition-related problems and behaviours of children and adolescents [9]. However, the number of scales available to assess children's nutritional literacy is limited and some of them have psychometric shortcomings [2]. Our results showed that the Turkish version of FNLQ-SC provided high levels of validity and reliability. The original scale consisted of 50 items (questions), 19 core components and 5 dimensions. The dimensions of knowledge and understanding, access to and planning for food, selecting food, preparing food and eating included 15, 5, 5, 10, 15 questions respectively. The overall FNLQ-SC questionnaire had acceptable internal consistency (Cronbach’s α = 0.698). The Cronbach’s α coefficients for the five dimensions (knowledge and understanding, access to and planning for food, selecting food, preparing food, eating), were 0.452, 0.300, 0.244, 0.148, and 0.436, respectively [20]. EFA was performed to determine the structure of the Turkish version of FNLQ-SC. EFA indicated that the FNLQ-TSC had three factorial structures that accounted for 33.14% of the total variance. However, six components (component 2, 7, 9, 10, 13, and 16) loaded on more than one factor and the difference between these factor loading values was less than 0.10. Therefore, these components were excluded from the scale by considering them as overlapping components, and the EFA analysis was re-run. EFA indicated that the FNLQ-TSC had three factorial structures (Fig. 1) that accounted for 42.0% of the total variance. The Cronbach’s α values for internal consistency were 0.679, 0.581, 0.551, and 0.350 for the FNLQ-TSC total score, factor-1, factor-2, and factor-3, respectively. FNLQ-TSC questionnaire had acceptable internal consistency. The Pearson correlation coefficients between each component and the overall questionnaire ranged from 0.134 to 0.430. The dimensional structure of the FNLQ-TSC obtained in the EFA was controlled by CFA. The three-factor model (Fig. 2) showed acceptable goodness-of-fit indices. (χ2/df = 1.924, RMSEA = 0.052, CFI = 0.864, GFI = 0.949). The correlation coefficients between each dimension and the overall questionnaire ranged from 0.300 to 0.842, especially the coefficients of factor-1 and factor-3, were more than 0.6, which showed a strong correlation with the overall questionnaire (Table 4).

The Turkish version of FNLQ-SC showed positive correlations with age, grade of class and discussion nutrition information with families, and negative correlation being only child. Gender was also an important variable affecting FNLQ-SC scores. Our findings are consistent with studies showing that nutritional literacy is associated with age, gender, education level, number of children and parental education level [20, 37]. The increase in nutritional literacy with age may be due to an increased interest in nutrition and health information and increased exposure to it [38]. Also younger children are not expected to develop the same level of complex skills as older teens or adults [35]. Many studies have shown that girls' nutritional literacy is higher than boys' [12, 39, 40]. This result may be a result of girls' higher interest in nutritional value and healthy nutrition [41] and their awareness that nutrition is an important component of health. Girls felt more empowered than boys to choose and control food and dietary choices, but may be less empowered to actually do so [42]. Parents education level and nutrition literacy, which may be an important educational target for improving child food and nutrition literacy. There were higher food and nutrition literacy among school-age adolescents who had a high father's education level, discussed nutrition information with their family in this study. Home food environment was significantly correlated with children’s food and nutrition literacy [20]. Parents can be role models in accessing and interpreting food and nutrition information, and in teaching children how to critically analyze the credibility and validity of information that shapes their nutritional knowledge [10, 43]. We also found a moderately positive and statistically significant relationship between the FNLQ-TSC total score and the HEI-2015 total score. Consistent with our results, studies showed that higher food literacy and nutrition were positively associated with healthier diet quality in children and adolescents [1, 44, 45]. Food and nutrition literacy questionnaires included many dimensions of food and nutrition knowledge; understanding, access, selection, preparation of food and healthy eating [46]. So food and nutrition literacy may significantly predict diet quality and nutrient density in adolescents (47).

Nevertheless, there are some limitations to our study. The relatively small sample size was one of the limitations of this study. Generalizability of study results may be limited to populations in similar areas with similar demographics. More factors that may affect the nutritional literacy of children and adolescents could have been considered. For example, it was not asked whether children had received any education about nutrition or foods. Nonetheless, we believe that the present study may shed light on future studies.

## Conclusion

The results suggested an acceptable validity and reliability of the Turkish version of FNLQ-SC questionnaire to measure food and nutrition literacy in school aged adolescents in Turkiye. And also the Turkish version of FNLQ-SC was significantly correlated with age, gender, grade of class, being an only child, discussing nutrition information with families and diet quality. It can be used to evaluate food and nutrition literacy in similar settings and age groups.

## Data Availability

The datasets generated and/or analyzed during the current study are not publicly available due we stated to the participants and parents that the data will not be shared with anyone for any purpose other than this study.
